# MEKK1, JNK, and SMAD3 mediate CXCL12-stimulated connective tissue growth factor expression in human lung fibroblasts

**DOI:** 10.1186/s12929-018-0421-9

**Published:** 2018-03-02

**Authors:** Chien-Huang Lin, Chung-Huang Shih, Yu-Chang Lin, You-Lan Yang, Bing-Chang Chen

**Affiliations:** 1 Graduate Institute of Medical Sciences, College of Medicine, Taipei Medical University, Taipei, Taiwan; 2 School of Respiratory Therapy, College of Medicine, Taipei Medical University, Taipei, Taiwan; 3 Division of Pulmonary Medicine, Department of Internal Medicine, Taipei Medical University Hospital, Taipei, Taiwan; 4 Department of Internal Medicine, School of Medicine, College of Medicine, Taipei Medical University, Taipei, Taiwan

**Keywords:** CXCL12, CTGF, Pulmonary fibrosis, Lung fibroblasts, SMAD3

## Abstract

**Background:**

In idiopathic pulmonary fibrosis, the interaction of CXCL12 and CXC receptor 4 (CXCR4) plays a critical role in lung fibrosis. Connective tissue growth factor (CTGF) overexpression underlies the development of pulmonary fibrosis. Our previous report showed that the Rac1-dependent extracellular signal-regulated kinase (ERK), c-Jun N-terminal kinase (JNK), and activator protein (AP)-1 pathways are involved in CXCL12-generated CTGF expression in human lung fibroblasts (WI-38). In present study, we additionally inspected the involvement of mitogen-activated protein kinase kinase kinase 1 (MEKK1)/JNK-dependent SMAD3 in CXCL12-triggered CTGF expression in WI-38 cells.

**Methods:**

WI-38 cells were stimulated with CXCL12 in the absence or presence of specific inhibitors or small interfering RNAs (siRNAs). CTGF expression and signaling transduction molecules were assessed by Western blot, luciferase activity assay, or ChIP assay.

**Results:**

CXCL-12-induced CTGF expression was attenuated by SIS3 (a SMAD3 inhibitor) and SMAD3 siRNA, but not by SB431542 (an activin receptor-like kinase 5, ALK5, inhibitor). CXCL12-stimulated CTGF expression was also attenuated by MEKK1 siRNA. Treatment of cells with CXCL12 caused an increase in SMAD3 phosphorylation at Ser208, translocation to nuclei, SMAD3-luciferase activity, and recruitment of SMAD3 to the CTGF promoter. Stimulation of cells with CXCL12 resulted in increase in JNK phosphorylation at Thr183/Tyr185 and MEKK1 phosphorylation at Thr261. Moreover, CXCL12-mediated SMAD3 phosphorylation or SMAD3-luciferase activity was inhibited by MEKK1 siRNA or SP600125. Finally, CXCL12-mediated JNK phosphorylation was attenuated by MEKK1 siRNA.

**Conclusion:**

In conclusion, results of this study suggest that CXCL12 activates the MEKK1/JNK signaling pathway, which in turn initiates SMAD3 phosphorylation, its translocation to nuclei, and recruitment of SMAD3 to the CTGF promoter, which ultimately induces CTGF expression in human lung fibroblasts.

## Background

Chronic lung diseases such as chronic obstructive asthma and idiopathic pulmonary fibrosis (IPF) are characterized by airway inflammation, accumulation of fibroblasts/myofibroblasts, aberrant remodeling of the lung architecture by excessive production of the type I collagen-rich matrix, and pulmonary fibrosis [1, 2]. One of the key processes in pulmonary fibrosis is the activation of fibroblasts into myofibroblasts [3], a process that seems to be dependent on activation of the activator protein (AP)-1 and SMAD3 pathway [4, 5]. The consensus is that myofibroblasts are ultimately responsible for the excessive deposition of the extracellular matrix (ECM) in pulmonary fibrosis. Resident fibroblasts express little connective tissue growth factor (CTGF); conversely, the CTGF is overexpressed by transforming growth factor (TGF)-β and cytokines that mediate pulmonary fibrosis [6–8].

CXCL12 acts on CXC receptor 4 (CXCR4) receptors to promote fibrocyte homing to the lungs and further lead to pulmonary fibrosis [9]. The chemokine, CXCL12 is critical to bone marrow (BM) stem cell development. Murine embryos lacking stromal cell-derived factor-1 (SDF-1) show multiple lethal defects, including impaired BM lymphoid and myeloid hematopoiesis [10]. A previous report indicated high levels of CXCL12 and CXCR4 in lung tissues of patients with IPF and interstitial pneumonia [11]. A previous study demonstrated that AMD3100 (a CXCR4 antagonist) attenuates bleomycin-produced lung fibrosis in mice [12]. Moreover, an anti-CXCL12 antibody inhibited bleomycin-induced fibrocyte migration to the lungs and reduced collagen deposition and α-smooth muscle actin (α-SMA) formation [13]. In general, CXCL12 triggers CXCR4 to activate G protein-coupled signaling pathways including mitogen-activated protein kinase (MAPK), which subsequently induce several cellular responses including gene expression and pulmonary fibrosis [14, 15]. A previous report indicated that CXCL12 mediates survival and metastasis of osteosarcomas through the c-Jun N-terminal kinase (JNK) signaling pathway [16]. Our previous study indicated that CXCL12/CXCR4-mediated CTGF expression is mediated by the activator protein (AP)-1 signaling pathway in human lung fibroblasts (WI-38) [17], but little information is available about the role of SMAD3 in CXCL12-stimulated CTGF expression in WI-38 cells.

CTGF was considered as a predictive indicator in pulmonary fibrotic disorders and also a prospective candidate in antifibrotic treatment [18, 19]. Earlier articles established that CTGF is highly expressed in a wide range of fibrotic conditions [20, 21]. Several studies pointed out that overexpression of CTGF contributes to the promotion of ECM accumulation and fibroblastic differentiation in tissue repair [7, 22, 23]. Therefore, overexpression of CTGF shows a vital role in tissue fibrosis. Several reports indicated that *ctgf* gene promoter region contains numerous transcription factor binding sites such as nuclear factor (NF)-κB, signal transducer and activator of transcription (STAT), activator protein-1 (AP-1), and SMAD [22–25]. Our previous studies demonstrated that AP-1 is involved in thrombin- and CXCL12-stimulated CTGF expression in WI-38 cells [8, 17]. At present, whether SMAD3 contributes to CXCL12-produced CTGF expression in lung fibroblasts is still unknown.

Mammalian c-Jun N-terminal kinase (JNK) is one major subfamily of mitogen-activated protein kinases (MAPKs), which are activated by a broad variety of stimuli including inflammatory mediators and growth factors [26]. Upon stimulation, mitogen-activated protein kinase kinase kinase 1 (MEKK1) mediates JNK phosphorylation and activation, which contributes to connective tissue remodeling by fibroblasts [27]. It was shown that JNK controls AP-1 activity that ultimately regulates the expression of fibrotic protein and lung fibrosis [17]. There is evidence that thrombin- or endothelin-1-induced CTGF expression is mediated through JNK activation in human lung fibroblasts [7, 8]. Previous report indicated that MEKK1 and JNK participated in thrombin-stimulated interleukin (IL)-8/CXCL8 expression in A549 cells [28, 29]. Nevertheless, the role of MEKK1 in regulating CXCL12-induced activations of JNK and SMAD3, and CTGF expression in WI-38 cells is still unclear. In this report, we revealed that CXCL12 triggers MEKK1 and JNK activation, which in turn initiate SMAD3 phosphorylation, SMAD3 transactivation, and recruitment of SMAD3 to the CTGF promoter, and ultimately induce CTGF expression in human lung fibroblasts.

## Methods

### Materials

CXCL12 was obtained from Peprotech (Rocky Hill, NJ, USA). SMAD3 small interfering (si)RNA [a mixture containing two specific SMAD3 siRNAs, catalog no. SAS10304006-004 (SMAD3: SASI_Hs01_00208931/SASI_Hs02_00340511)], MEKK1 siRNA [a mixture containing two specific MEKK1 siRNAs, catalog no. SAS10107011-001 (MEKK1 SASI_Hs02_00340548/551)], control siRNA [con siRNA, 5’-GAU CAU ACG UGC GAU CAG A-3′ (sense)], SIS3, SB431542, and an antibody specific for α-tubulin (catalog no. T5168) were obtained from Sigma (St. Louis, MO, USA). SP600125 was purchased from Calbiochem-Novabiochem (San Diego, CA, USA). Lipofectamine Plus reagent, Lipofectamine 2000 reagent, penicillin/streptomycin, fetal calf serum (FCS), and Minimum essential medium (MEM) were obtained from Invitrogen Life Technologies (Carlsbad, CA, USA). Antibodies specific for JNK phosphorylated at Thr183/Tyr185 (catalog no. 9251), JNK (catalog no. 9252), and SMAD3 (catalog no. 9523) were obtained from Cell Signaling Technology (Beverly, MA, USA). Antibodies specific for SMAD3 phosphorylated at Ser208 (catalog no. sc-130,218), CTGF (catalog no. sc-14,939), and rabbit polyclonal immunoglobulin G (IgG) (catalog no. sc-66,931), and anti-mouse (catalog no. sc-2005), anti-rabbit (catalog no. sc-2004), and anti-goat (catalog no. sc-2020) IgG-conjugated horseradish peroxidase (HRP) were acquired from Santa Cruz Biotechnology (Santa Cruz, CA, USA). MEKK1 phosphorylated at Thr261 antibody (catalog no. A8129) was obtained from Assay Biotech (Sunnyvale, CA, USA). An antibody specific for MEKK1 (catalog no. GTX46204) was purchased from GeneTex (Irvine, CA, USA). A chromatin immunoprecipitation (ChIP) assay kit was acquired from Upstate Biotechnology (Lake Placid, NY, USA). pBK-CMV-*Lac Z* (*LacZ*) was provided by professor W-W. Lin (National Taiwan University, Taipei Taiwan). All materials for sodium dodecylsulfate polyacrylamide gel electrophoresis (SDS-PAGE) were purchased from Bio-Rad (Hercules, CA, USA). All other chemicals were obtained from Sigma.

### Cell culture

WI-38 cells, a normal human embryonic lung fibroblast cell line, were purchased from American Type Culture Collection (Manassas, VA, USA), and cell cultures were prepared as described previously [17]. In brief, WI-38 cells were grown in an MEM growth medium containing 10% FCS in a humidified 37 °C incubator with 5% CO_2_. Cells were used between passages 18 and 30 for all experiments. After reaching confluence, cells were seeded onto 6-cm dishes for cell transfection and immunoblotting, onto 12-well plates for the cell transfection and luciferase assays, onto 10-cm dishes for the ChIP assay, and onto 4-well culture slides for immunofluorescence staining and confocal microscopy.

### Western blot analysis

Western blot analysis were presented as previously described [17]. In brief, cells were stimulated with CXCL12, pretreated with SP600125, SIS3, or SB431542 for 30 min, or transfected with SMAD3 siRNA, MEKK1 siRNA, or control siRNA using Lipofectamine 2000 for 24 h before they were stimulated with CXCL12. Immunoreactivity was revealed using Western blot analysis. Quantitative data were achieved by a computing densitometer using a scientific imaging system (Eastman Kodak, Rochester, NY, USA).

### Transfection and CTGF- or SMAD3-luciferase assay

The luciferase activity assay was as previously described [17]. In brief, WI-38 cells (5 × 10^4^ cells/well) were transfected with Lipofectamine Plus with CTGF-Luc, SMAD3-Luc, and 0.5 μg of *Lac Z* for 6 h. The medium was changed with basal medium without FCS for 18 h. The cells were treated with CXCL12 for additional 16 h, and then luciferase activity assay method was used. To examine the influences of c-Jun siRNA or SMAD3 siRNA in CXCL12-induced CTGF-luciferase activity, WI-38 cells were co-transfected with c-Jun siRNA, SMAD3 siRNA, CTGF-Luc, and *Lac Z* for 24 h before they were exposed to CXCL12. To assay the effects of JNK in CXCL12-induced SMAD3-luciferase activity, SP600125 was added to cells for 30 min before the addition of CXCL12. The degree of generation of luciferase activity was calculated as the ratio of cells with and those without treatment.

### Immunofluorescence staining and confocal microscopy

Immunofluorescence staining and confocal microscopy were defined in our previous report [4]. Briefly, WI-38 cells were stimulated with CXCL12 (10 ng/ml) for indicated time intervals. Slides were blocked with 5% bovine serum albumin (BSA) and incubated with antibodies specific to SMAD3 for 2 h, after which it was incubated with a Alexa Fluor 488-conjugated secondary antibody for another 1 h. The slides were stained with DAPI to visualize nuclei. SMAD3 was detected using a confocal fluorescence microscope (Leica TCS SP5, Wetzlar, Germany).

### ChIP assay

Human lung fibroblasts were treated with 10 ng/ml CXCL12 for 20 min and subsequently fixed by formaldehyde for further 10 min. The SMAD3 binding to CTGF promoter region was achieved using a ChIP assay as we described previously [17]. Polymerase chain reaction (PCR) augmentations of SMAD3 on the CTGF promoter area were presented using the following primers: SMAD3, 5′-AGT GGT GCG AAG AGG ATA GG-3′ (sense) and 5′-CAT TCC TCG CAT TCC TCC CC-3′ (antisense). Extracted DNA (2 μl) was used for 38 cycles of amplification in a 50 μl reaction mixture under the following conditions: 95 °C for 30 s, 60 °C for 60 s, and 72 °C for 30 s. PCR products were analyzed by 2% agarose gel electrophoresis.

### Statistical analysis

All data passed the normality test. The results are displayed as the mean ± S.E.M. based on at least three independent experiments. A one-way analysis of variance (ANOVA) was achieved and followed by Dunnett’s test to determine the difference between groups. Values of *p* < 0.05 were deliberated statistically significant.

## Results

### Involvement of SMAD3, but not ALK5, in CXCL12-induced CTGF expression

**Fig. 1:**
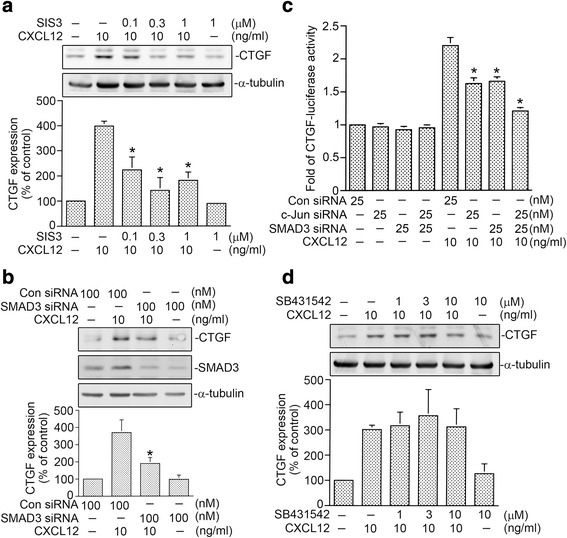
SMAD3 mediates CXCL12-induced connective tissue growth factor (CTGF) expression in human lung fibroblasts (WI-38). **a** WI-38 cells were treated with 0.1~ 1 μM of SIS3 for 20 min before cells were exposed to CXCL12 (10 ng/ml) for additional 2 h. Protein levels of CTGF or α-tubulin in cell lysates were determined by western blot. Statistics are showed as the mean ± S.E.M. of three separate experiments. * *p* < 0.05, compared with CXCL12 treatment without SIS3. **b** After transfected with control siRNA (Con siRNA 100 nM) or SMAD3 siRNA (100 nM) for 24 h, WI-38 cells were exposed to CXCL12 (10 ng/ml) for additional 2 h. Protein levels of CTGF, SMAD3, or α-tubulin in cell lysates were determined by western blot. Statistics are showed as the mean ± S.E.M. of three separate experiments. * *p* < 0.05, compared with CXCL12 treatment with control siRNA. **c** After transfected with control siRNA (Con siRNA 25 nM), c-Jun siRNA (25 nM), SMAD3 siRNA (25 nM), 0.5 μg CTGF-Luc, and 0.2 μg of pBK-CMV-Lac Z for 24 h, the WI-38 cells were exposed to CXCL12 (10 ng/ml) for additional 24 h. The CTGF-luciferase activity assay is described in “Materials and Methods”. Statistics are showed as the mean ± S.E.M. of four separate experiments. * *p* < 0.05, compared to CXCL12 treatment alone. **d** WI-38 cells were treated with 1~ 10 μM of SB431542 for 20 min before cells were exposed to CXCL12 (10 ng/ml) for additional 2 h. Protein levels of CTGF or α-tubulin in cell lysates were determined by western blot. Statistics are showed as the mean ± S.E.M. of three separate experiments. * *p* < 0.05, compared with CXCL12 treatment without SB431542

Numerous reports revealed that CXCL12 is an effective chemoattractant for fibrocytes that leads to airway fibrosis [30, 31]. Additionally, CTGF also contributes to tissue fibrosis [7, 22, 23]. Many studies revealed that CTGF expression is regulated by activation of several transcription factors, including AP-1 and SMAD3 [22, 24]. Previous report indicated that AP-1 activation is involved in CXCL12-stimulated CTGF expression [17]. Our previous data revealed that 3~ 30 mg/ml CXCL12 produced CTGF expression, with a highest effect at 10 ng/ml in WI-38 cells. To further explore whether SMAD3 is participated in CXCL12-stimulated CTGF expression in WI-38 cells, a SMAD3 inhibitor (SIS3) and SMAD3 siRNA were used. We found that SIS3 (0.1~ 1 μM) inhibited CXCL12-stimulated CTGF expression. When WI-38 cells were treated with 1 μM SIS3, CXCL12-stimulated CTGF expression was inhibited by 54 ± 9% (Fig. 1a). Moreover, siRNA experiments revealed that SMAD3 siRNA (100 nM) reduced CXCL12-stimulated CTGF expression by 48 ± 4% (Fig. 1b, upper panel). To check the effect of SMAD3 siRNA experiment, we utilized SMAD3 siRNA to inhibit the SMAD3 protein expression in WI-38 cells. As a result, we found that SMAD3 siRNA markedly inhibited SMAD3 protein expression in WI-38 cells (Fig. 1b, middle panel). To verify whether concomitant knockdown c-Jun and SMAD3 can further down-regulate CTGF-luciferase activity, c-Jun siRNA and SMAD3 siRNA were used. We found that c-Jun siRNA (25 nM) and SMAD3 siRNA (25 nM) both inhibited CXCL12-stimulated CTGF-Luciferase activity by 47 ± 7% and 39 ± 5%, respectively. Moreover, combination of c-Jun siRNA and SMAD3 siRNA further attenuated CXCL12-stimulated CTGF-luciferase activity by 78 ± 3%. (Fig. 1c). A previous report demonstrated that ALK5-dependent SMAD3 activation is participated in TGF-β-stimulated CTGF expression in hepatic stellate cells [32]. To determine whether ALK5 participates in CXCL-12-stimulated CTGF expression, an ALK5 inhibitor SB431542 was used. We found that SB431542 (1~ 10 μM) did not affect CXCL12-induced CTGF expression (Fig. 1d). These results suggest that AP-1 and SMAD3, but not ALK5, are involved in CXCL12-induced CTGF expression in WI-38 fibroblasts.

### CXCL12 induced SMAD3 activation in human lung fibroblasts

**Fig. 2:**
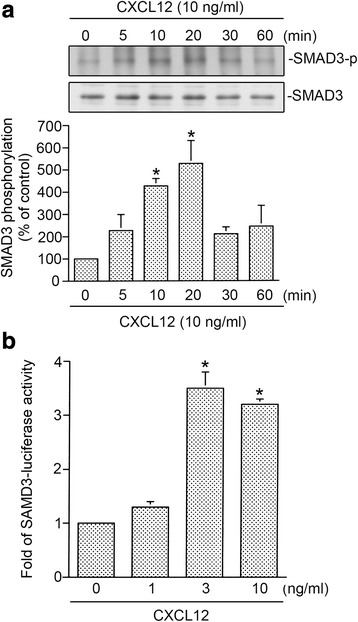
CXCL12-induced SMAD3 phosphorylation and SMAD3-lucifease activity in human lung fibroblasts (WI-38). **a** WI-38 cells were exposed to 10 ng/ml of CXCL12 for 0~ 60 min. Protein levels of phospho-SMAD3 Ser208 and SMAD3 in cell lysates were determined by western blot. Statistics are showed as the mean ± S.E.M. of three separate experiments. * *p* < 0.05, compared with control without CXCL12 treatment. **b** Transfection of WI-38 cells with 0.5 μg of SMAD3-Luc and 0.2 μg of pBK-CMV-Lac Z for 24 h. The WI-38 cells were exposed to CXCL12 (1~ 10 ng/ml) for additional 16 h. Luciferase activity assay were described in “Materials and Methods”. Statistics are showed as the mean ± S.E.M. of three separate experiments. * *p* < 0.05, compared with control without CXCL12 treatment

**Fig. 3:**
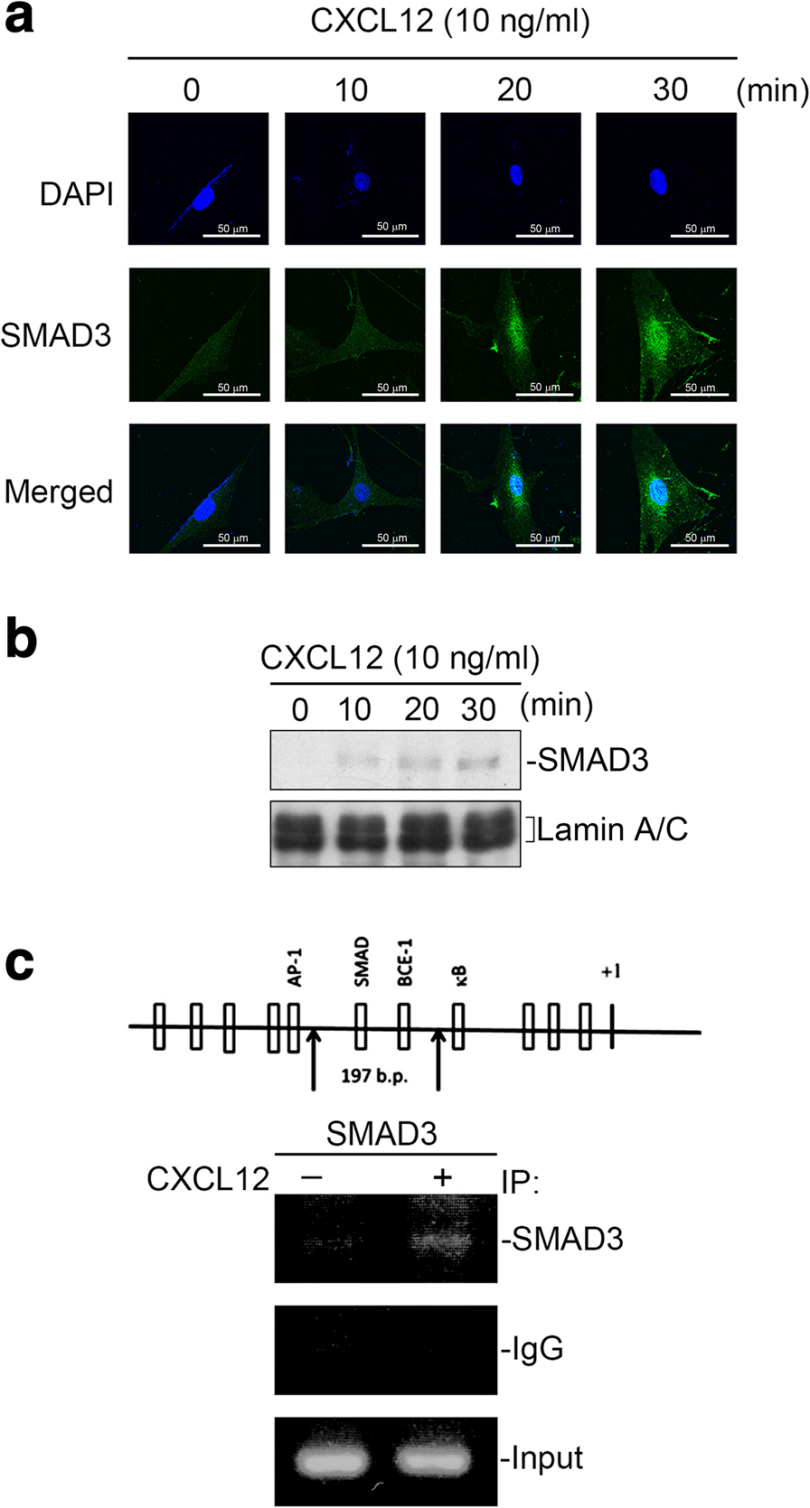
CXCL12-induced SMAD3 nuclear translocation and recruitment of SMAD3 on the connective tissue growth factor (CTGF) promoter in WI-38 cells. **a** WI-38 cells were exposed to 10 ng/ml of CXCL12 for 0~ 30 min. In a confocal microscopic image analysis (200×), cells were marked with SMAD3, and nuclei were specified by DAPI. Slides are illustrative of similar results from three separate experiments. **b** Cells were exposed to 10 ng/ml of CXCL12 for 0~ 30 min. Nuclear protein were prepared and immunodetected with specific antibodies for SMAD3 or lamin A/C. Traces are illustrative of three separate experiments. **c** Cells were exposed to 10 ng/ml of CXCL12 for 20 min and identified using ChIP assay as defined in “Materials and Methods”. PCR amplification using primers designed against the SMAD3-binding site was performed. Identical volumes of soluble cross-linked chromatins existing in each PCR were approved by the product for input. Negative control: A rabbit polyclonal IgG antibody. Typical traces are illustrative of similar results from three separate experiments

As mentioned above, SMAD3 is participated in CXCL12-stimulated CTGF expression in WI-38 cells. We further examined whether SMAD3 activation is involved in the signaling cascade of CXCL12-induced CTGF expression. Phosphorylation of the Ser208 residue in SMAD3 caused functional activation [33], and the antibody against phosphorylated Ser208 was used to examine SMAD3 activation. As shown in Fig. 2a, WI-38 cells were stimulated with CXCL12 (10 ng/ml) for 0~ 30 min, SMAD3 Ser208 phosphorylation had raised at 5 min, peaked at 20 min, and decreased after 30 min. To directly determine SMAD3 activation after CXCL12 treatment, WI-38 cells were transiently transfected with SMAD3-Luc as an indicator of SMAD3 activation. Stimulation of WI-38 cells with CXCL12 (1~ 10 ng/ml) produced an increase in SMAD3-luciferase activity (Fig. 2b). Yang et al. [34] reported that SMAD3 translocated into nuclei and then increased profibrogenic gene expression. We used immunofluorescence staining and confocal microscopy to test this result. As shown in Fig. 3a, CXCL12 induced SMAD3 translocation from the cytosol to nuclei after 20~ 30 min of stimulation. Western blot experiments revealed that CXCL12 stimulated SMAD3 translocation into the nucleus during 10~ 30 min of treatment (Fig. 3b). Next, to explore whether SMAD3 is binding to the human CTGF promoter region after CXCL12 stimulation, ChIP assay was used. We found that CXCL12 produced an increase in recruitment of SMAD3 to the SMAD3 binding site of the human CTGF promoter region (Fig. 3c). Taken together, these results suggest that the activation of SMAD3 plays a role in CXCL12-induced CTGF expression in human lung fibroblasts.

### JNK mediates CXCL12-induced SMAD3 phosphorylation and SMAD3-luciferase activity

**Fig. 4:**
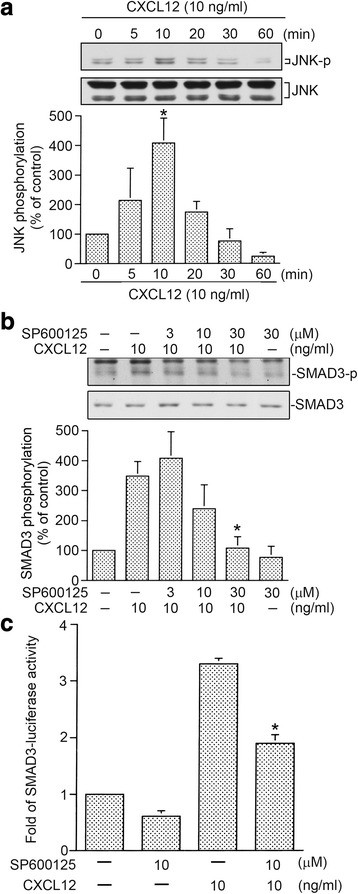
Involvement of c-Jun N-terminal kinase (JNK) in CXCL12-induced SMAD3 activation in human lung fibroblasts (WI-38). **a** Cells were exposed to 10 ng/ml of CXCL12 for 0~ 60 min. Protein levels of phospho-JNK T183/Y185 or JNK in cell lysates were determined by western blot. Statistics are showed as the mean ± S.E.M. of three separate experiments. * *p* < 0.05, compared with control without CXCL12 treatment. **b** WI-38 cells were treated with 3~ 30 μM of SP600125 for 20 min before cells were exposed to CXCL12 (10 ng/ml) for additional 20 min. Protein levels of phospho-SMAD3 Ser208 and SMAD3 in cell lysates were determined by western blot. Statistics are showed as the mean ± S.E.M. of three separate experiments. * *p* < 0.05, compared with CXCL12 treatment without SP600125. **c** After transfected with 0.5 μg of SMAD3-Luc and 0.2 μg of pBK-CMV-Lac Z for 24 h, the WI-38 cells treated with 10 μM of SP600125 for 20 min before cells were exposed to CXCL12 (10 ng/ml) for additional 16 h. Luciferase activity assay were described in Fig. 2b. Statistics are showed as the mean ± S.E.M. of three separate experiments. * *p* < 0.05, compared with CXCL12 treatment

JNK can be activated by a variety of stimuli including thrombin and CXCL12 [8, 35]. Previous report showed that JNK is involved in CXCL12-mediated CTGF expression in WI-38 cells [17]. In this report, we further examined whether CXCL12 can stimulate phosphorylation of JNK. Fig. 4a shows that treating cells with CXCL12 (10 ng/ml) resulted in the time-dependent JNK Thr183/Tyr185 phosphorylation. The JNK phosphorylation reached a maximum at 10 min after CXCL12 stimulation (Fig. 4a). Previous report indicated that TGF-β1-produced JNK activation regulates SMAD3 phosphorylation in rat peritoneal mesothelial cells [36]. Next, we further studied whether CXCL12-caused SMAD3 phosphorylation and AMAD3-luciferase activity occur through JNK signal pathway. Fig. 4b shows that SP600125 (3~ 30 μM) reduced CXCL12-caused SMAD3 Ser208 phosphorylation in a concentration-dependent manner. SP600125 (30 μM) inhibited CXCL12-caused SMAD3 phosphorylation by 70 ± 11% (Fig. 4b). Similarly, SP600125 (10 μM) also inhibited CXCL12-stimulated SMAD3-luciferase activity by 44 ± 7% (Fig. 4c). Taken together, this result indicates that JNK is an upstream kinase of SMAD3 in CXCL12-stimulated responses in lung fibroblasts.

### MEKK1 mediated CXCL12-caused CTGF expression

**Fig. 5:**
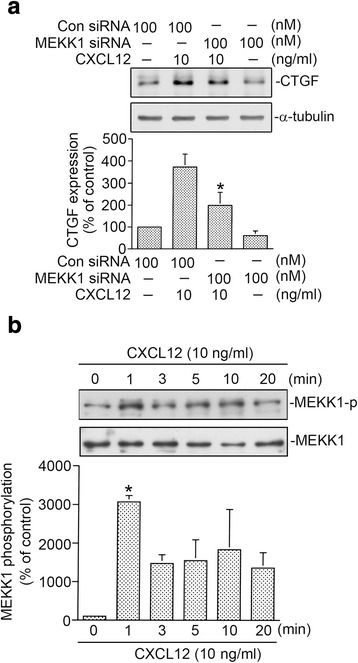
Involvement of mitogen-activated protein kinase kinase kinase 1 (MEKK1) in CXCL12-caused connective tissue growth factor (CTGF) expression in WI-38 cells. **a** After transfected with control siRNA (Con siRNA 100 nM) or MEKK1 siRNA (100 nM) for 24 h, the WI-38 cells were exposed to CXCL12 (10 ng/ml) for additional 2 h. Protein levels of CTGF or α-tubulin in cell lysates were determined by western blot. Statistics are showed as the mean ± S.E.M. of four separate experiments. * *p* < 0.05, compared with CXCL12 treatment without MEKK1 siRNA. **b** WI-38 cells were exposed to CXCL12 (10 ng/ml) for 0~ 20 min. Protein levels of phospho-MEKK1 Thr261 or MEKK1 were determined by western blot. Statistics are showed as the mean ± S.E.M. of three separate experiments. * *p* < 0.05, compared with control without CXCL12 treatment

Our previous report revealed that MEKK1 mediated hypoxia-stimulated CTGF expression in WI-38 cells [4]. In an attempt to determine whether MEKK1, an upstream of JNK, partakes in CXCL12-caused CTGF expression, MEKK1 siRNA was used. Fig. 5a shows that MEKK1 siRNA (100 nM) reduced CXCL-12-mediated CTGF expression by 49 ± 11% (Fig. 5a). The Thr261 phosphorylated residue in MEKK1 results in an increase in its enzymatic activation [37]. Next, to examine whether CXCL12 can induce an increase in MEKK1 phosphorylation, the phosphorylated MEKK1 Thr261 antibody was used. We found that CXCL12 caused an increase in MEKK1 Thr261 phosphorylation (*n* = 3) (Fig. 5b). Taken together, these data suggest that MEKK1 activation mediates CXCL12-caused CTGF expression in WI-38 cells.

### Involvement of MEKK1 in CXCL12-caused JNK and SMAD3 phosphorylation

**Fig. 6:**
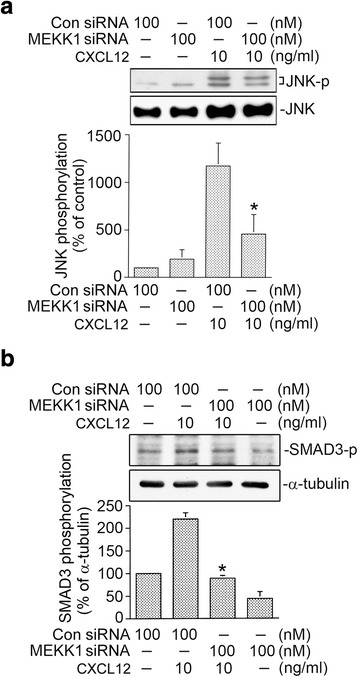
Mitogen-activated protein kinase kinase kinase 1 (MEKK1) mediates CXCL12-stimulated c-Jun N-terminal kinase (JNK) phosphorylation and SMAD3 phosphorylation in WI-38 cells. **a** After transfected with control siRNA (Con siRNA 100 nM) or MEKK1 siRNA (100 nM) for 24 h, the WI-38 cells were exposed to CXCL12 (10 ng/ml) for additional 10 min. Protein levels of phospho-JNK T183/Y185 or JNK in cell lysates were determined by western blot. Statistics are showed as the mean ± S.E.M. of three separate experiments. * *p* < 0.05, compared with CXCL12 treatment without MEKK1 siRNA. **b** After transfected with control siRNA (Con siRNA 100 nM) or MEKK1 siRNA (100 nM) for 24 h, the WI-38 cells were exposed to CXCL12 (10 ng/ml) for additional 20 min. Protein levels of phospho-SMAD3 Ser208 or α-tubulin in cell lysates were determined by western blot. Statistics are showed as the mean ± S.E.M. of three separate experiments. * *p* < 0.05, compared with CXCL12 treatment without MEKK1 siRNA

We further explored the role of MEKK1 in CXCL12-caused JNK Thr181/Tyr185 phosphorylation and SMAD3 Ser208 phosphorylation. Fig. 6a shows that MEKK1 siRNA (100 nM) reduced CXCL12-caused JNK Thr181/Tyr185 phosphorylation by 75 ± 19% (Fig. 6a). Moreover, we found that MEKK1 siRNA also diminished CXCL12-stimulated SMAD3 Ser208 phosphorylation by 59 ± 3% (Fig. 6b). These findings suggest that MEKK1 occurs upstream of JNK and SMAD3 in CXCL12-caused CTGF expression in human lung fibroblasts.

## Discussion

Our findings demonstrated that CXCL12 activates the MEKK1 and JNK signaling pathways, which in turn initiates SMAD3 activation and induces CTGF expression in human lung fibroblasts. A growing body of evidence indicated that CTGF plays a pathological role in lung diseases, and several lines of evidence suggest that CTGF is also a profibrotic mediator of pulmonary fibrotic diseases such as IPF [7, 38]. Recent studies established that CXCL12/CXCR4 axis characterizes the key pathway in regulation of fibrocyte migration and participates in pulmonary fibrosis [12, 39]. Moreover, the levels of CXCL12 were increased in the plasma of IPF patients and were correlated with numbers of circulating fibrocytes [40]. Several reports showed that treatment of mice with a CXCL12 antibody or CXCR4 antagonist (AMD3100) diminished bleomycin-stimulated lung fibrosis [12, 41]. These data indicated that CXCL12 or CTGF plays a critical role in lung fibrosis. Our previous paper revealed that CXCL12 provoked expression of CTGF via the Rac/ERK, JNK, and AP-1 pathways [17]. In the present report, we offer an explanation of additional mechanism via which CXCL12 causes activation of MEKK1/JNK to induce SMAD3 phosphorylation and binding to the CTGF promoter, and finally causes expression of CTGF in human lung fibroblasts. Our findings display two pathways related with CXCL12 and CTGF, and offer the development of therapeutic approaches to diminish pulmonary fibrosis of IPF triggered by CXCL12.

SMADs comprise a family of eight structurally related transcription factors that play critical roles in regulating gene expression [42]. The canonical SMAD pathway is the chief mediator of TGF-β signaling [42]. SMAD3 was reported to play a critical role in pulmonary fibrosis through inducing expressions of several profibrotic mediators including CTGF [43]. In addition, a previous report indicated that the G protein-coupled receptor, such as angiotensin II, activates SMAD3 via a TGF-β/ALK5-independent pathway [44]. Multiple signaling pathways have been related to *CTGF* gene expression. Our previous data revealed that Rac1/ERK-dependent AP-1 activation participated in CXCL12-caused CTGF expression in WI-38 cells [17]. In addition, a previous study showed that activation of SMAD also participates in lysophosphatidic acid (LPA)-stimulated CTGF expression in gingival fibroblasts [45]. This paper exhibited that SMAD3 activation participated in CXCL12-stimulated CTGF expression in human lung fibroblasts. This is based on the fact that CXCL12-stimulated CTGF expression was inhibited by a SMAD3 inhibitor (SIS3). Moreover, SMAD3 siRNA reduced CXCL12-generated CTGF expression. Moreover, CXCL-12 produced SMAD3 phosphorylation at Ser208. Furthermore, CXCL12 induced SMAD3 binding to the CTGF promoter region. Our findings are consistent with a previous report which revealed that SMAD3 participates in TGF-β-stimulated CTGF expression in human lung fibroblasts [46]. Moreover, a previous research paper exhibited that TGF-β enhances CXCL12-induced chemotaxis and homing of naive T cells [47]. Several studies indicated that CTGF induction by LPA requires transactivation of ALK5 in mouse skeletal muscles and human gingival fibroblasts [45, 48]. In contrast, a previous article indicated that angiotensin II mediates CTGF expression via SMAD phosphorylation, which is a TGF-β-independent mechanism [44]. In present report, SB431542 (an ALK5 inhibitor) did not affect CXCL12-caused CTGF expression. Chambers et al. also indicated that ALK5 is not involved in thrombin-induced CTGF expression in human lung fibroblasts [49]. Therefore, these results suggest that SMAD3, but not ALK5, is involved in CXCL12-caused CTGF expression in WI-38 cells.

Several reports revealed that MEKK1 was shown to activate MAPK including JNK signaling pathways [50]. A previous report found that MEKK1 and MEK are involved in CTGF expression by various stimuli [6, 51]. Leask et al. presented that MEKK1 mediates TGF-β-caused CTGF expression in human skin fibroblasts [6]. Additionally, several reports demonstrated that the JNK participated in the induction of CTGF expression by various stimuli in WI-38 cells [7, 8]. Our previous studies demonstrated that endothelin-1-, thrombin-, and CXCL12-caused CTGF expression require JNK activation in human lung fibroblasts [7, 8, 17]. Yang et al. [43] reported that TGF-β induced CTGF expression via JNK activation in human gingival fibroblasts. In this paper, we found that the MEKK1 siRNA repressed CXCL12-caused CTGF expression. Additionally, CXCL12 induces JNK phosphorylation at Thr183/Tyr185, which is inhibited by MEKK1 siRNA. Taken together, we suggest that the MEKK1/JNK signaling pathway participates in CXCL12-stimulated CTGF expression in human lung fibroblasts.

Transcriptional activity of SMAD3 may be regulated by multiple phosphorylation sites including Ser208. SMAD3 phosphorylated at linker regions or COOH-terminal regions exists as separate molecules with different functions and transmits distinct signals [52]. In addition to recruiting SMAD3 to the CTGF promoter, cyclic tensile strain appears to regulate the activity of SMAD3 through phosphorylation of chondrocytic cells [53]. It is well known that the activation of MAPK such as by JNK and cyclin-dependent kinase 4 is required for growth factor-induced phosphorylation of SMAD3 [54, 55]. A previous study demonstrated that JNK participated in SMAD3 Ser208 phosphorylation, and this phosphorylation contributes to the SMAD3 activation and causes cancer progression [56]. Given that JNK plays a critical role in SMAD3 activity, we speculated that MEKK1 and JNK might be upstream of SMAD3 by CXCL12 stimulation, and we examined this possibility. We found that SP600125 attenuated CXCL12-caused SMAD3-luciferase activity. Moreover, CXCL12-mediated SMAD3 phosphorylation was attenuated by MEKK1 siRNA and SP600125. Accordingly, these data suggest that CXCL12-mediated SMAD3 activation is controlled by the MEKK1/JNK cascade in human lung fibroblasts.

## Conclusions

**Fig. 7:**
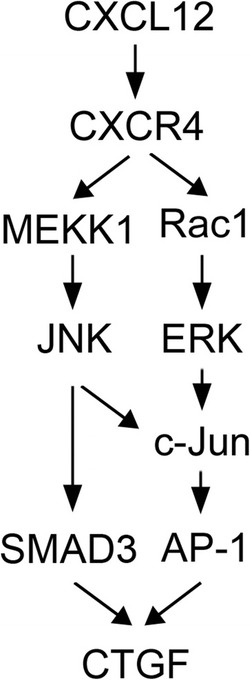
Graphic brief shows that how signal pathway of CXCL12 stimulates expression of connective tissue growth factor (CTGF) in human lung fibroblasts. It was previously revealed that the Rac1/extracellular signal-regulated kinase (ERK)-dependent activator protein (AP)-1 pathway participated in CXCL12-caused expression of CTGF in WI-38 cells. In this report, CXCL12 also activated the mitogen-activated protein kinase kinase kinase 1 (MEKK1) signaling pathway, which turned on c-Jun N-terminal kinase (JNK) and SMAD3 activation and finally caused expression of CTGF in human lung fibroblasts (WI-38)

In conclusion, the current study together with our previous study [17] indicates that treatment of human lung fibroblasts with CXCL12 induced CTGF expression via the Rac1/ERK, JNK, and AP-1 and MEKK1/JNK/SMAD3 signaling pathways. This study showed that CXCL12 triggers MEKK1/JNK activation, which sequentially begins SMAD3 phosphorylation at Ser208, and its binding to the CTGF promoter, causing CTGF expression in WI-38 cells. Fig. 7 is an illustration revealing that CXCL12 stimulated CTGF expression through Rac1/ERK, JNK, AP-1, and MEKK1/JNK/SMAD3 in human lung fibroblasts.

## Abbreviations

ALK5: Activin receptor-like kinase 5
AP-1: Activator protein-1
BM: Bone marrow
ChIP: Chromatin immunoprecipitation
CTGF: Connective tissue growth factor
CXCR4: CXC receptor 4
ECM: Extracellular matrix
ERK: Extracellular signal-regulated kinase
FCS: Fetal calf serum
HRP: Horseradish peroxidase
IgG: Immunoglobulin G
IPF: Idiopathic pulmonary fibrosis
JNK: c-Jun N-terminal kinase
LPA: Lysophosphatidic acid
MAPK: Mitogen-activated protein kinase
MEKK1: Mitogen-activated protein kinase kinase kinase 1
MEM: Minimum essential medium
NF-κB: Nuclear factor-κB
PCR: Polymerase chain reaction
SDF-1: Stromal cell-derived factor-1
SDS-PAGE: Sodium dodecylsulfate polyacrylamide gel electrophoresis
siRNA: Small interfering RNA
STAT: Signal transducer and activator of transcription
TGF-β: Transforming growth factor-β
α-SMA: α-smooth muscle actin
